# Pregnancy Outcomes in Women With Primary Adrenal Insufficiency: Data From a Multicentre Cohort Study

**DOI:** 10.1111/1471-0528.18143

**Published:** 2025-03-30

**Authors:** Matthew Cauldwell, Philip J. Steer, Masato Ahsan, Amanda Ali, Shabana Ashiq, Rebecca Ashworth, Deena Basha, Hsu Chong, Gillian A. Corbett, Fidelma Dunne, Amanda Hill, Katarzyna Gajewska‐Knapik, Adam Jakes, David McLaren, Therese Kinsella, Tara Lee, Miles Levy, Lucy MacKiliop, Fionnuala M. McAuliffe, Aarthi Mohan, Clare Mumby, Melanie Nana, Catherine Napier, Francesca Neuberger, Christine Newman, Tabitha Oosterhouse, Amelia Shard, Hassan Shehata, Linden Stocker, Jeremy W. Tomlinson, Adele Beck, Bijay Vaidya, Kate Wiles, Catherine Williamson, Julia Zollner, Emma Ward, Helen E. Turner

**Affiliations:** ^1^ Department of Obstetrics, Maternal Medicine Service St George's Hospital London UK; ^2^ Academic Department of Obstetrics and Gynaecology Chelsea and Westminster Hospital London UK; ^3^ Department of Endocrinology University Hospitals Leicester UK; ^4^ Department of Obstetrics Kingston Hospital London UK; ^5^ Department of Obstetrics Liverpool Women's Hospital Liverpool UK; ^6^ Department of Maternal Medicine Epsom and St Helier Hospital Carshalton UK; ^7^ Academic Department of Obstetrics, Birmingham Women's and Children's NHS Foundation Trust University of Birmingham Birmingham UK; ^8^ UCD Perinatal Research Centre, School of Medicine, University College Dublin National Maternity Hospital Dublin Ireland; ^9^ Department of Diabetes Obesity and Endocrinology Galway University Hospital, Clinical Trial Network in Diabetes, Institute for Clinical Trials University of Galway Galway Ireland; ^10^ Maternal Medicine Department North Bristol NHS Trust Bristol UK; ^11^ Rosie Maternity Unit, Department of Obstetrics Addenbrookes Hospital Cambridge UK; ^12^ Leeds Centre for Diabetes and Endocrinology Leeds Teaching Hospitals NHS Trust Leeds UK; ^13^ Department of Obstetrics, St Michael's Hospital University Hospitals Bristol NHS Foundation Trust Bristol UK; ^14^ Department of Obstetrics Norfolk and Norwich University Hospitals Norfolk UK; ^15^ Nuffield Department of Women's and Reproductive Health University of Oxford Oxford UK; ^16^ St. Mary's Hospital, Manchester University Foundation Trust Manchester UK; ^17^ Guy's and St Thomas' NHS Foundation Trust London UK; ^18^ Department of Endocrinology Newcastle upon Tyne Hospitals Newcastle upon Tyne UK; ^19^ Department of Obstetrics, Ysbyty Gwynedd Betsi Cadwaldar University Health Board Saint Asaph UK; ^20^ Department of Obstetrics University Hospitals Southampton Southampton UK; ^21^ Department of Endocrinology, Oxford Centre for Diabetes, Endocrinology and Metabolism Oxford University Hospitals NHS Foundation Trust Oxford UK; ^22^ Department of Endocrinology, Royal Devon & Exeter Hospital University of Exeter Medical School Exeter UK; ^23^ Department of Maternal Medicine Barts Health NHS Trust London UK; ^24^ Department of Obstetrics University College Hospital London UK

**Keywords:** adrenal crisis, pregnancy, primary adrenal insufficiency

## Abstract

**Objective:**

To determine characteristics and pregnancy outcomes in women with primary adrenal insufficiency (PAI).

**Design:**

Retrospective multicentre cohort study.

**Setting:**

Twenty‐three maternity units in the UK and Ireland.

**Sample:**

Seventy‐nine women with PAI who had 101 pregnancies.

**Method:**

Retrospective chart analysis.

**Main Outcome Measures:**

Adrenal crisis, pregnancy outcomes.

**Results:**

We obtained data on 101 pregnancies in 79 women with PAI. Most (51, 64.1%) had autoimmune disease, 8 (10.3%) had prior adrenal infarction/surgery/haemorrhage, 2 (2.6%) had congenital adrenal hyperplasia, and 18 (21.3%) were unclassified. 19 (24%) women experienced a crisis during pregnancy (18.8% of pregnancies). One woman died postpartum. Although all women had recorded endocrinology input during pregnancy, steroid emergency cards were only reportedly carried in 40 (39.6%) pregnancies and 9/19 (47.4%) of those with an adrenal crisis in pregnancy. Compared with the pre‐pregnancy dose, only 41% of women received an increased hydrocortisone dose in pregnancy. The caesarean section rate was higher than the UK average: 62/97 (63.9%). The preterm birth rate was 21.2% (21/99) and 12.8% (12/94) of neonates had a birthweight < 10th centile.

**Conclusion:**

Whilst the obstetric outcome of pregnancy with PAI is generally favourable, there are high rates of caesarean birth and prematurity. A high number of women experienced adrenal crisis and further exploration is warranted. Recommendations regarding third trimester increases in hydrocortisone need consideration and potentially strengthening, in light of further evidence. Pregnant women with adrenal insufficiency should carry an NHS steroid warning card; this should be reinforced both by endocrine and obstetric teams because of the increased risk of life‐threatening adrenal crisis.

## 1 Introduction

Primary adrenal insufficiency (PAI) is a potentially life‐threatening endocrine disorder. The most common cause is autoimmune destruction of the adrenal cortex (‘autoimmune Addison's disease’) which accounts for 70% of cases in high‐income countries [1]. Other causes include adrenal haemorrhage and neoplastic disease. The reported prevalence is around 300 cases per million [1, 2]. PAI is defined by lack of cortisol production from the adrenal cortex due to disease/destruction of the adrenal gland. This usually requires life‐long glucocorticoid replacement therapy, with dose adjustment at times of acute illness and physical stress. Deficiency of mineralocorticoid and adrenal androgens also necessitates replacement of mineralocorticoid in the majority of patients. PAI is more prevalent in females compared with males with peak incidence in the reproductive years. Despite data from a US population based study that reported a recent doubling of the prevalence of Addison's disease (AD) in pregnancy from 5.6 per 100 000 in 2003 to 9.6 per 1 000 000 in 2011 [3], there are relatively few large contemporary studies describing pregnancy outcomes in women with PAI. A 2024 report suggested that there are increased rates of caesarean delivery, small for gestational age (SGA) babies and miscarriage [4]. Studies also indicate an increased risk of preterm birth and adrenal crisis [4, 5, 6, 7, 8]. Maternal mortality secondary to PAI is reported to be uncommon [9]; however, the 2023 MBBRACE report describes two maternal deaths where PAI was sub optimally managed [10].

Guidance on PAI and pregnancy is largely based on expert consensus with little published evidence on optimal hydrocortisone dose replacement in pregnancy [11, 12]. Based on physiological increases in total and free cortisol concentrations during pregnancy, standard guidance suggests increasing hydrocortisone doses by 20%–40% in the third trimester, though this is not based on prospective data [12]. Any increased dose should have minimal fetal effect as glucocorticoid is metabolised by the placenta [13]. There are few recommendations regarding mineralocorticoid replacement requirements, despite the physiological changes in pregnancy. Aldosterone increases during pregnancy, and progesterone is an aldosterone antagonist [14]. Thus, fludrocortisone may also need to be increased in the third trimester [9] but is less commonly modified in pregnancy.

In order to improve understanding of maternal and fetal outcomes in PAI and pregnancy and to assess current practice management, we conducted a UK multicentre audit where data on management and outcomes for both mother and baby were captured. The objective of the study was to determine the characteristics and outcomes of pregnancy in women with PAI. Given the recent MBBRACE data on maternal deaths related to the disorder, we also collected data on maternal education regarding self‐management of glucocorticoid replacement and the occurrence of adrenal crisis in pregnancy.

## 2 Patient Involvement

There was no direct patient involvement in this study.

## 3 Methods

UK and Ireland centres that have a combined obstetric endocrine clinic were contacted via email in May 2023 to participate in a retrospective audit of pregnancies in women with PAI. Additionally, the Society for Endocrinology advertised the audit online through their website from May 2023 until February 2024. Twenty‐three sites were able to provide data. Each centre collected data from pregnancies in women with PAI identified from January 2013 until March 2024 (median number 4, range one to seven). Women with AI were divided into four separate groups—(1) Autoimmune disease, (2) Previous adrenal surgery/infarction/haemorrhage, (3) Congenital Adrenal Hyperplasia, and (4) Unknown. Women with secondary and tertiary adrenal insufficiency were excluded.

Demographic data and pregnancy outcomes up to six weeks postpartum were collected by chart review by the local investigators and then collated by the lead investigator into a single dataset for analysis using SPSS V.29 for Windows (IBM, Armonk, NY, USA). To ensure correction of any transcription errors, data were downloaded from the combined SPSS dataset into Excel by centre and verified by each centre individually (no individual data were shared between centres). We assessed pre‐pregnancy dosing of glucocorticoids and fludrocortisone, as well as the presence of maternal hypothyroidism or pre‐existing diabetes mellitus. Where women were taking prednisolone, we converted this to the equivalent dosage of hydrocortisone, using a ratio of 1 mg of prednisolone to 4 mg of hydrocortisone [15]. We obtained information on patient education and evidence of specialist review, as well as documented crises in the year prior to pregnancy/during pregnancy, as well as the underlying cause. We assessed pregnancy complications and co‐morbidities that included chronic hypertension, gestational hypertension and pre‐eclampsia. Data were collected on gestation and mode of birth, birthweight and birthweight centile. We also recorded whether there was a documented episode of neonatal unit admission. We collected maternal biochemistry data where available to assess serum sodium and potassium concentrations in each trimester.

Data were analysed using SPSS V.29 for Windows (IBM, Armonk, NY, USA). Categorical data are presented as frequencies (numbers) and percentages. Data are presented as medians with the interquartile range (IQR). Correlations were calculated using Pearson's product moment correlation if variables were continuous and Spearman's rank‐order correlation if either variable was ordinal. Differences between continuous variables were assessed with the Wilcoxon signed rank test. All tests were two‐tailed and *p* < 0.05 were considered statistically significant.

This audit was approved by the research audit committee at St George's Hospital London in October 2022, and individual centres registered audits locally.

## 4 Results

A total of 23 maternity units contributed data to this study. Data were collected from 111 pregnancies in 88 women, but in 10 pregnancies, there were insufficient data for meaningful analysis, resulting in 101 pregnancies from 79 women being available for analysis. Fifty‐one (64.1%) women had autoimmune disease and had 66 (65.3%) pregnancies; 8 (10.3%) women with adrenal infarction/surgery/haemorrhage had 10 (9.9%) pregnancies; 2 (2.6%) women with congenital adrenal hyperplasia had 2 pregnancies, while 18 (21.3%) women with unclassified PAI had 23 (22.8%) pregnancies. Two women had 3 pregnancies recorded, 18 had two, and 59 had only one. 57 (56.4%) pregnancies were nulliparous. The women's demographics at their first recorded pregnancy are shown in Table 1. 77 (94.9%) of women were White European, one was South Asian, and one was ‘other’ ethnic group. Forty‐one of 72 (56.9%) women with data on BMI were overweight (BMI > 25 kg/m^2^). Hydrocortisone was the predominantly used glucocorticoid, and prednisolone was only used in three cases (two women with autoimmune and one women was unknown).

**TABLE 1:** Demograhic data at first recorded pregnancy (*N* = 79).

Variable	Missing	Mean (SD)	Median	IQR	Minimum	Maximum
Age at diagnosis (years)	12	24.7 (8.6)	26.0	19.0–30.0	1	40
Age at pregnancy (years)	2	33.2 (5.1)	34.0	30.5–36.0	22	48
Height (cm)	8	165 (6.8)	165	161–169	149	181
Weight (kg)	8	70.5 (16.3)	64.0	59.0–78.0	49	116
BMI (kg/(mm^a^))	7	26.2 (6.3)	24.0	21.7–29.8	17	41

^a^ Results to three significant figures. In some cases, only the BMI was recorded, not both height and weight.

All subsequent analyses include pregnancies where data are available (maximum *N* = 101). 89/101 (88.1%) pregnancies were conceived spontaneously and 12 (11.9%) pregnancies through in vitro fertilisation. 98/101 (97%) pregnancies were singleton and there were 3 sets of twins. Pregnancy outcome data are reported in Table 2. Mode of delivery was not reported in 4 pregnancies. Including twin pregnancies, 32 (33.0%) were delivered by elective caesarean, 30 (30.9%) by emergency caesarean and 35 (36.1%) had a vaginal birth (including instrumental deliveries). 21/99 (21.2%) babies with data on gestational age at birth were born preterm (< 37 completed weeks of gestation). 12/94 (12.8%) babies were SGA (< 10th centile) at delivery. 17/94 (18.1%) babies where data were available were admitted to the neonatal unit (data not recorded in 7 pregnancies). 11 (64.7%) of the babies admitted to the neonatal unit were preterm and 6 (35.3%) were term.

**TABLE 2:** Outcome of pregnancy, excluding three twin pregnancies (*N* = 98).

Variable	Missing	Mean (SD)	Median	IQR	Minimum	Maximum
Duration of pregnancy (weeks)	2	37.8 (2.22)	38.0	37.0–39.0	27	41
Birthweight (g)	6	3230 (674)	3297	2803–3707	901	4510
Birthweight centile	8	57.5 (33.1)	62.5	26.5–89.0	1	99
Estimated blood loss (mL)	14	613.0	500.0	400–661	200	2360

Two (1.98%) pregnancies were classified as being complicated by chronic hypertension, 6 (5.9%) by gestational hypertension and 10 (9.9%) by pre‐eclampsia. Ten pregnancies were complicated by gestational diabetes mellitus. None of these complications correlated with the dosage of hydrocortisone or fludrocortisone.

**FIGURE 1:**
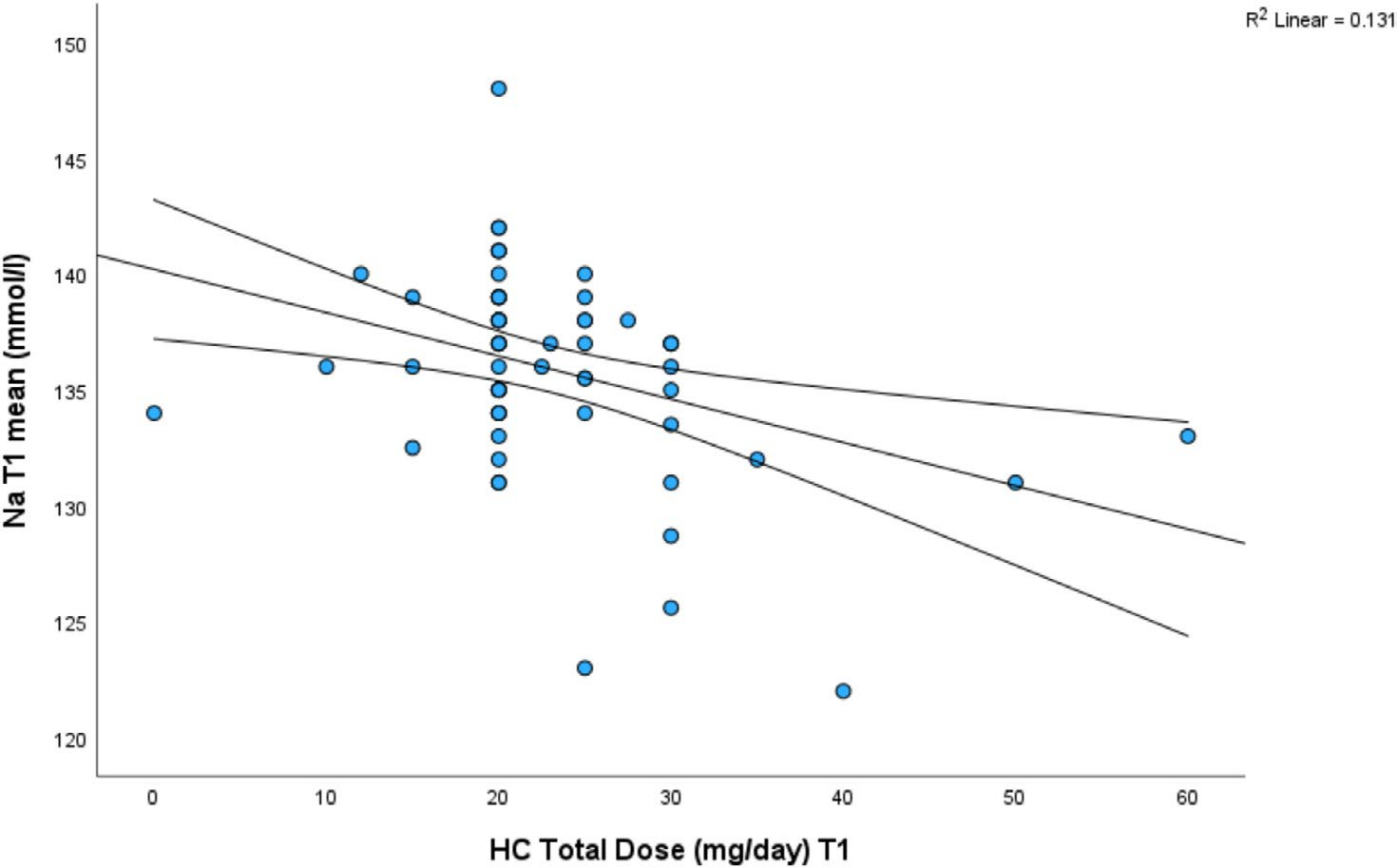
Correlation between hydrocortisone total daily dose in the first trimester and blood Na levels.

**FIGURE 2:**
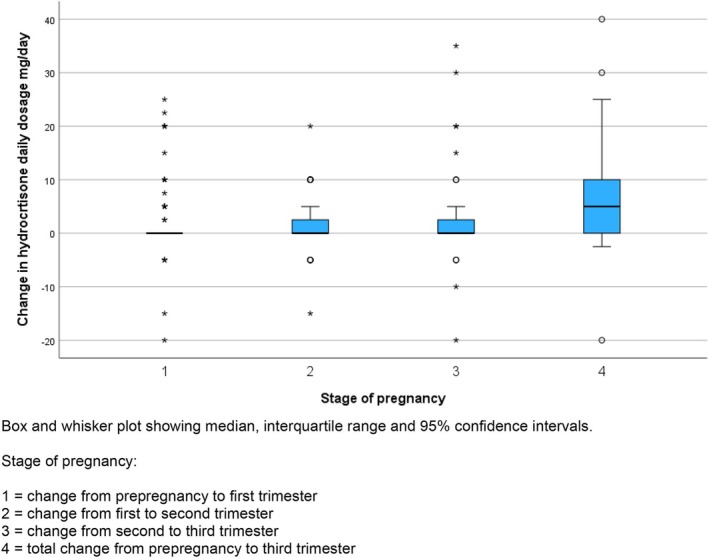
Changes in total daily hydrocortisone dose by stage of pregnancy.

The dosages of hydrocortisone used by pregnancy stage are shown in Figure 1 and Table S1. In 67/94 (71.3%) pregnancies with hydrocortisone dosage, there was no change in the daily total dose prescribed between pre‐pregnancy and 1st trimester; in 22 (23.4%) there was an increase in dose (mostly of 5 mg (7 cases, 7.4%) or 10 mg (5 cases, 5.3%), maximum 25 mg) and in 5 (5.3%) pregnancies there was a decrease (maximum decrease 20 mg). Between the first and second trimesters, 65/95 (68.4%) of doses remained unchanged; 26 (27.3%) were increased (17 (17.9%) by 5 mg (max 20 mg)), and 4 (4.2%) were decreased. Between the second and third trimesters, 65/94 (69.1%) doses were unchanged; 25 (26.5%) were increased (14 (14.9%) by 5 mg, maximum 35 mg) and doses were decreased in 4 (4.3%, maximum 20 mg in one patient considered to be on a high dose of hydrocortisone pre‐pregnancy). When compared with the pre‐pregnancy dose, the dose in the third trimester had not changed in 42/93 (45.2%) and increased in 39 (41.9%)—(in 22 (23.7%) by 5 mg, in 11 (11.8%) by 10 mg and 4 (4.3%) each by 15 and 20 mg; the maximum increase was 40 mg). In two cases, the dose was reduced (by 2.5 and 20 mg). The changes are graphically summarised in Figure 2.

**FIGURE 3:**
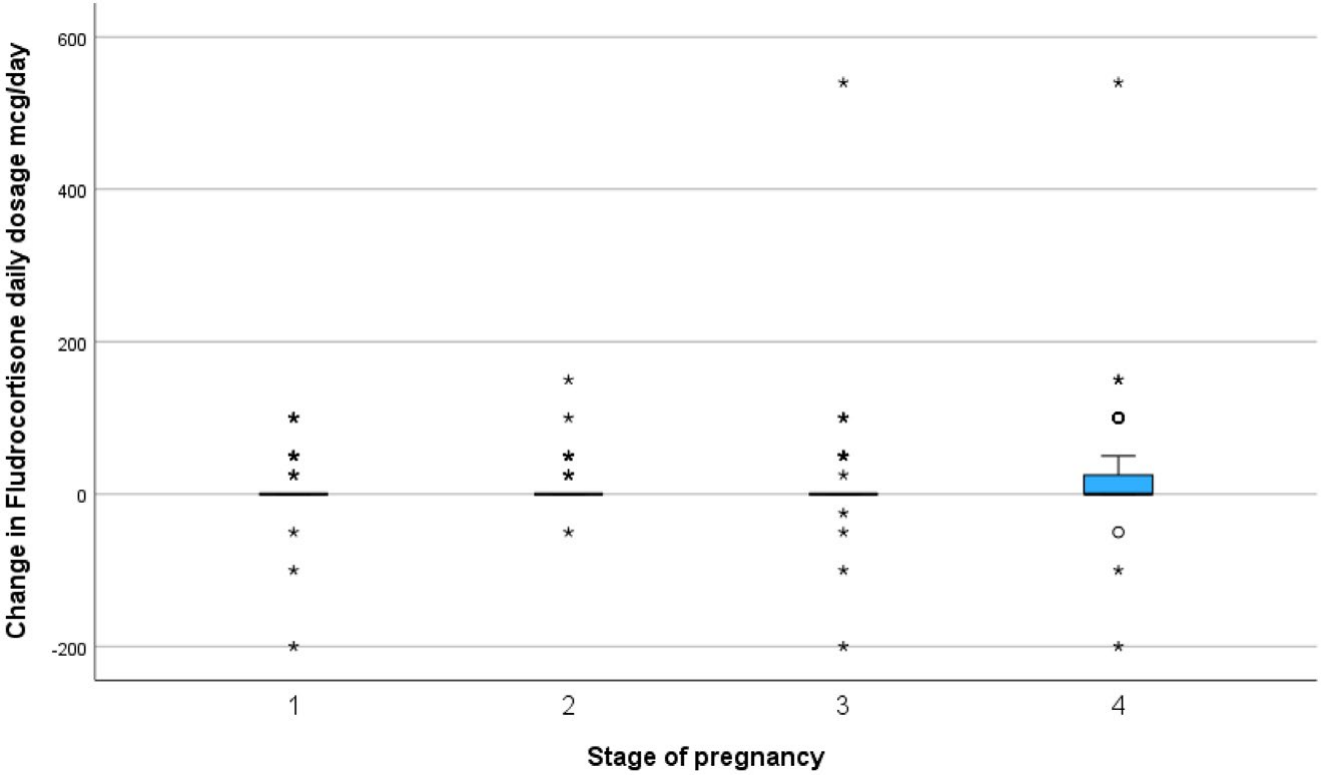
Changes in total daily fludrocortisone dose by stage of pregnancy. Box and whisker plot showing median, interquartile range and 95% confidence intervals. Stage of pregnancy: 1 = change from prepregnancy to first trimester. 2 = change from first to second trimester. 3 = change from second to third trimester. 4 = total change from prepregnancy to third trimester.

**FIGURE 4:**
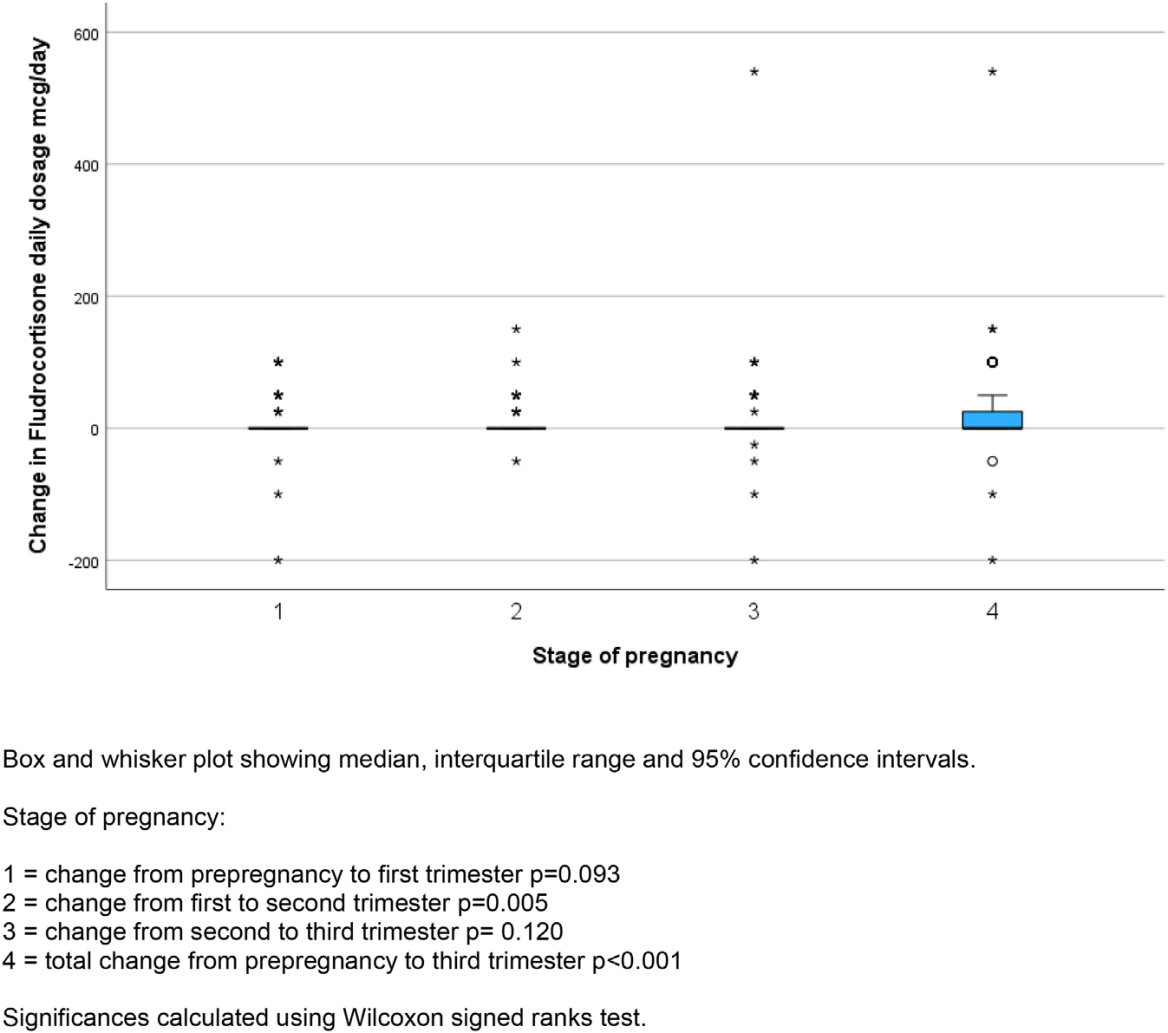
Changes in total daily fludrocortisone dose by stage of pregnancy.

Prior to pregnancy (61/65) 95.8% of women with autoimmune disease, 5/9 (44.4%) of women with adrenal haemorrhage/infarction/surgery, 1/2 (50.0%) of women with congenital adrenal hyperplasia, and 14/20 (70%) of those with undefined PAI received fludrocortisone treatment. The dosages of fludrocortisone are shown by pregnancy stage in Figure 3 and Table S2. In 83/96 (83.3%) pregnancies with fludrocortisone dosage, there was no change in the total daily dose prescribed between pre‐pregnancy and 1st trimester, in 13(13.5%) there was an increase in dose (25 mcg in 3 (3.1%), 50 mcg in 6 (6.3%) and 100 mcg in 4 (4.2%)). Between the first and second trimesters, the dose was unchanged in 83/97 (85.6%), increased in 13 (10%) (4 by 25 mcg, 7 by 50 mcg and one each by 100 mcg and 150 mcg) and decreased in 4 (maximum by 50 mcg). Between the second and third trimesters, 81/96 (84.4%) dosages remained unchanged; in 11 (11.5%) there was an increase (one (1%) by 25 mcg, 6 (6.3%) by 50 mcg, 3 (3.1%) by 100 mcg and one (1%) by 540 mcg) and 4 decreased (one each by 25, 50, 100 and 200 mcg). When compared with the pre‐pregnancy dose, the dose in the third trimester had not changed in 64/95 (67.4%), increased in 28 (29.5%)—in 5 (5.3%) by 25 mcg, in 11 (11.6%) by 50 mcg and in 9 (9.5%) by 100 mcg; the maximum increase was 540 mcg. In three cases, the dose was reduced (by 50, 100 and 200 mcg). The changes are summarised in Figure 4.

Nine women had a documented adrenal crisis in the year prior to the pregnancy, 8 had one and one woman had 3 (11.4% of women, 8.9% of pregnancies). During pregnancy, 19 women (24% of women, 18.8% of pregnancies) had a documented crisis; only 2 (10.5%) of these had a history of a crisis in the year pre‐pregnancy. Crises were treated with incremental dose increases of hydrocortisone dosage. Crises were attributed to hyperemesis in 9/19 (47.4%) cases, urinary tract infection in 5/19 (26.3%) cases and presumed gastroenteritis in 2/19 (10.5%) cases. In 3/19 (15.8%) cases no clear cause was documented. More than 50% (11/19) of crises occurred in the first trimester, but not all cases (5/19) recorded the gestation the crisis occurred. Confirmed carriage of an ‘NHS Steroid Emergency Card’ was documented in only 40 of all pregnancies (39.6%), and in only 9 of the 19 (47.4%) having a crisis in pregnancy, despite all women having specialist endocrine review in pregnancy. Only 5/19 of the women with adrenal crisis in pregnancy increased hydrocortisone dose in the third trimester. There were no maternal deaths during pregnancy but one woman died within 6 weeks of delivery from an adrenal crisis.

The mean and lowest serum sodium concentrations and mean and highest serum potassium concentrations by trimester are shown in Table S3 and show no correlation between pre‐pregnancy doses of hydrocortisone or fludrocortisone and electrolyte concentrations during pregnancy. Nor was there any significant correlation between hydrocortisone dose and electrolyte levels during pregnancy except for the dose and sodium concentration in the first trimester *p* = 0.003 (Figure S1). The lack of any other correlations suggests that this may have occurred by chance.

## 5 Discussion

### 5.1 Main Findings

Our study presents contemporary detailed data from a relatively large cohort of women managed in more than 15 UK and Ireland centres. It includes data from women with autoimmune disease but also with PAI of different aetiologies. Pregnancy complications in this cohort of women were uncommon, with rates of gestational hypertension and pre‐eclampsia similar to the background UK rates [16]. However, it is of concern to note the rate of reported adrenal crisis, the low number of women recorded as carrying a steroid alert card and the death of one woman following pregnancy.

Rates of preterm delivery in our study were higher (21.2%) than the England and Wales average of 7.9% in 2022 [17]; but similar to the rate of 21% reported from a recent European multicentre study of women with adrenal insufficiency [3]. It has been hypothesised that because women with PAI have a disorder of the hypothalamic–pituitary–adrenal axis, this will contribute to a greater rate of iatrogenic preterm birth [18]. However, the precise mechanism of this is unclear. In our study, there was a higher than average rate of caesarean delivery (63.9%). The average rate of caesarean section for England in 2022 was 34.7% [19]. This finding is similar to other studies of women with PAI which also show high rates of caesarean birth [3]. It is unclear why rates of caesarean section are so high, although ‘medicalisation’ of this group of women is hypothesised, and in our cohort, women were older—median age at first recorded pregnancy was 33 years—and 56.9% of women had a BMI in excess of 25 in their first recorded pregnancy.

A total of 19 women had a crisis during pregnancy, giving an overall rate of 18.8%. This figure is more than double the number reported in a recent international study on Addison's in pregnancy [3]. It is possible that the increased rate of crisis may have been due to a failure to increase hydrocortisone dosages during pregnancy. However, 57.8%, the adrenal crisis occurred within the first trimester when routine increase in hydrocortisone dose is not standard practice. Nausea and vomiting affects at least 50% of pregnancies so it is unsurprising this was responsible for the majority of crises [15]. However, since first trimester nausea and vomiting are common, in future anticipation of this issue should be included when counselling women. Where nausea and vomiting impairs a pregnant woman's ability to take her prescribed corticosteroid replacement, she should be advised to seek urgent medical review. Managing crises in pregnancy can be clinically challenging and it is vital that those with adrenal insufficiency carry a steroid alert card, consider wearing an emergency alert necklace/bracelet and understand appropriate self‐management during intercurrent illness [19]. One of the maternal deaths in the recent MBBRACE Report was attributed to poorly managed nausea and vomiting during pregnancy [10]. Only 2/19 (10.5%) women who had a crisis during pregnancy were reported to have had a crisis in the year prior to pregnancy, highlighting the importance of patient education around the increased risk of adrenal crisis in pregnancy in all women with PAI. Only nine women had a documented crisis in the year prior to pregnancy; our data suggests, therefore, that pregnancy is a time when women who have had historically good control are more vulnerable to crisis and may require admission to hospital for treatment. This is important information that should be shared with women during preconception care. As NICE guidance [20] is clear regarding intrapartum management of steroids, we opted not to assess this in our study.

It was encouraging to note that all women were reviewed by a specialist during pregnancy (endocrinologist or maternal medicine physician); however, only relatively few women had documented evidence of carrying an NHS emergency steroid card during pregnancy. In the UK, steroid cards are recommended for all patients in PAI. In 2020, the Society of Endocrinology, in conjunction with NHS England, released NHS Emergency Steroid Card guidance [21]. These are intended to assist when patients are admitted and become seriously unwell and are unable to communicate their problems. Moreover, only 9/19 (47.4%) of the women who developed adrenal crisis pre‐pregnancy and during pregnancy were documented to have carried a steroid card. Thus, while PAI is rare, increased awareness for patients and those managing pregnancy remains of utmost importance. We also acknowledge that maternal death is rare but include one case where death occurred postpartum; this reinforces the importance of clinicians being aware that a crisis can occur at any point in pregnancy/postpartum and requires prompt treatment. The woman who died in our study presented post‐delivery with ongoing nausea and vomiting to a different hospital from where she received her specialist care during pregnancy. It was not recognised that she was having a crisis, and this was not promptly treated. Notably, this woman did increase her hydrocortisone in the third trimester and carried a steroid card, and had two crises during pregnancy.

### 5.2 Interpretation

In contrast to previous studies (and perhaps reflecting the small number of CAH in the cohort), all women were receiving hydrocortisone replacement, except for three who were prescribed prednisolone. The dose was very similar in all women and aligned with standard non‐pregnancy guidelines. Moreover, our study has illustrated that clinicians did not routinely increase hydrocortisone doses in pregnancy, particularly in the third trimester. This practice aligns with recent NICE guidance published in 2024, which states an increased dose of hydrocortisone should be considered dependent on clinical symptoms [22]. This is in contrast to the Endocrine Society guideline on pregnancy and AD, which places an emphasis on increasing hydrocortisone in the third trimester. In keeping with previous studies, there were instances where there was a dose reduction of hydrocortisone in pregnancy [3]. It was reassuring that in cases where there was an increase in hydrocortisone during pregnancy, there were no associated increases in the incidence of maternal hypertension, pre‐eclampsia or diabetes mellitus. Whilst current pregnancy guidance does not usually comment on fludrocortisone replacement and the possible need to increase it [12], it is recognised that a proportion of women do require increased doses related to the mineralocorticoid antagonistic effect of progesterone. Indeed, in the multicentre European study, almost a quarter of pregnancies required an increased dose of fludrocortisone. In our study, only 29.5% required an increase in dose from pre‐pregnancy to the third trimester.

It is reassuring that pregnant women with PAI have a generally favourable obstetric outcomes, and that the majority are managed according to standard NICE guidance. However, the high rate of reported crisis is important and more work is warranted to try to understand and prevent this. In addition to considering an increase in hydrocortisone in pregnancy, recent NICE guidance also recognises the possible need to increase fludrocortisone dosage depending on symptoms, serum sodium and postural blood pressure [22]. The reason for the higher‐thanthan‐average rate of Caesarean delivery is unclear but should be highlighted to patients during pre‐conceptual discussions. Crucially, pre‐conceptual awareness of patients and clinicians regarding steroid safety, improvement in awareness and education around adrenal crisis, and the need to seek urgent medical attention remain of utmost importance.

### 5.3 Strength and Limitations

The primary strength of this study is that data are presented from a relatively large number of pregnancies and include data on maternal and fetal outcomes. However, as this study was retrospective, there were many cases where data were not available, which potentially could impact results. We were unable to capture data on miscarriage rates, although previous studies have shown higher rates in women with PAI. Whilst we had hoped to analyse data on maternal biochemical parameters and study maternal serum sodium and potassium concentrations in each trimester, we did not have enough data to assess this clearly. We recommend that clinicians should continue to monitor maternal serum biochemistry during pregnancy to exclude hyponatremia and hyperkalemia. We were also limited in having full clinical details regarding the timing and precipitant of adrenal crisis in all cases; however, in the future, a further understanding of adrenal crisis precipitant in pregnancy would be helpful for patient education and clinician safety netting and management.

## 6 Conclusion

All women with PAI should have access to specialist preconception care to ensure comprehensive counselling. This should include a discussion about steroid safety, sick day rules, consideration of emergency alert identification jewellery, ensuring NHS emergency steroid alert cards are carried and reiterating the need for access to rapid treatment if a crisis is suspected. Whilst obstetric outcomes for women with PAI are generally favourable, the risk of crisis is increased during pregnancy, with an associated risk of mortality. Evidence‐based guidelines are required to understand the most appropriate glucocorticoid and fludrocortisone management during pregnancy. Care for women should be individualised, and current UK practice demonstrates this. All clinicians providing care for women with PAI in pregnancy should be aware of the increased risk of adrenal crisis, which can be life‐threatening.

## Author Contributions

M.C., H.E.T., P.J.S., L.M. and J.W.T. conceived the idea for the study. M.A., A.A., S.A., R.A., D.B., H.C., G.A.C., F.D., K.G.‐K., A.J., D.M., T.K., T.L., A.M., C.M., M.N., C.N., F.N., C.N., T.O., A.S., L.S., A.B., C.W., J.Z. and E.W. collated the data. P.J.S. analysed the data. The first draft was written by M.C., H.E.T. and P.J.S. All authors evaluated previous versions of the manuscript and have read and accepted the final version.

## Ethics Statement

This study was approved by St George's Hospital Audit Committee on 9th December 2022.

## Conflicts of Interest

L.M. is a part time employee of Optum UK.

## Supporting information


**Figure S1.** Supporting Information.

**Table S1.** Supporting Information.

**Table S2.** Supporting Information.

**Table S3.** Supporting Information.

## Data Availability

The data that support the findings of this study are available from the corresponding author upon reasonable request.
