# Supplementary material for: Pregnancy Outcomes in Women With Primary Adrenal Insufficiency: Data From a Multicentre Cohort Study
Source: BJOG. 2025 Mar 30;132(8):1122–9. doi: 10.1111/1471-0528.18143 (PMC12137789; doi:10.1111/1471-0528.18143)
Supplement: Supplementary file 1 — Figure S1. Supporting Information. [file BJO-132-1122-s002.docx]

Supplementary Figure 1. Correlation between hydrocortisone total daily dose in the first trimester and blood Na levels


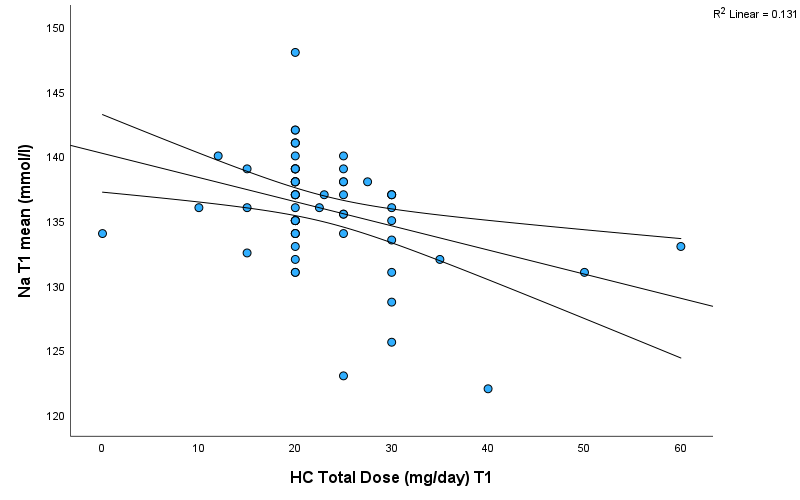


X axis: Hydrocortisone total dose (mg/day) in the first trimester

Y axis: Serum sodium concentration in the first trimester (mmol/l)
