# Supplementary material for: Pregnancy Outcomes in Women With Primary Adrenal Insufficiency: Data From a Multicentre Cohort Study
Source: BJOG. 2025 Mar 30;132(8):1122–9. doi: 10.1111/1471-0528.18143 (PMC12137789; doi:10.1111/1471-0528.18143)
Supplement: Supplementary file 2 — Table S1. Supporting Information. [file BJO-132-1122-s004.docx]

Table 1 Total daily dose of hydrocortisone (mg/day) in relation to pregnancy stage

**HC Total Dose (mg/day) prepregnancy**

|  | | Frequency | Percent |
| --- | --- | --- | --- |
| Valid | 0 | 2 | 2.0 |
|  | 10 | 1 | 1.0 |
|  | 12 | 1 | 1.0 |
|  | 15 | 9 | 8.9 |
|  | 16 | 1 | 1.0 |
|  | 18 | 1 | 1.0 |
|  | 20 | 56 | 55.4 |
|  | 23 | 1 | 1.0 |
|  | 23 | 1 | 1.0 |
|  | 24 | 1 | 1.0 |
|  | 25 | 14 | 13.9 |
|  | 30 | 7 | 6.9 |
|  | 35 | 1 | 1.0 |
|  | 40 | 1 | 1.0 |
|  | 60 | 1 | 1.0 |
|  | Total | 98 | 97.0 |
| Missing |  | 3 | 3.0 |

| **HC Total Dose (mg/day) First Trimester (0-14 weeks)** |
| --- |

|  | | Frequency | Percent |
| --- | --- | --- | --- |
| Valid | 0 | 1 | 1.0 |
|  | 10 | 1 | 1.0 |
|  | 12 | 1 | 1.0 |
|  | 15 | 3 | 3.0 |
|  | 16 | 1 | 1.0 |
|  | 18 | 2 | 2.0 |
|  | 20 | 52 | 51.5 |
|  | 23 | 2 | 2.0 |
|  | 25 | 13 | 12.9 |
|  | 28 | 1 | 1.0 |
|  | 30 | 10 | 9.9 |
|  | 35 | 1 | 1.0 |
|  | 40 | 4 | 4.0 |
|  | 44 | 1 | 1.0 |
|  | 50 | 1 | 1.0 |
|  | 60 | 1 | 1.0 |
|  | Total | 95 | 94.1 |
| Missing |  | 6 | 5.9 |

| **HC Total Dose (mg/day) Second Trimester (15-28 weeks)** |
| --- |

|  | | Frequency | Percent |
| --- | --- | --- | --- |
| Valid | 0 | 1 | 1.0 |
|  | 10 | 1 | 1.0 |
|  | 12 | 1 | 1.0 |
|  | 16 | 1 | 1.0 |
|  | 20 | 43 | 42.6 |
|  | 23 | 3 | 3.0 |
|  | 25 | 21 | 20.8 |
|  | 30 | 14 | 13.9 |
|  | 35 | 3 | 3.0 |
|  | 40 | 4 | 4.0 |
|  | 44 | 1 | 1.0 |
|  | 50 | 1 | 1.0 |
|  | 60 | 1 | 1.0 |
|  | Total | 95 | 94.1 |
| Missing |  | 6 | 5.9 |

| **HC Total Dose (mg/day) Third trimester (29 weeks and later)** |
| --- |

|  | | Frequency | Percent |
| --- | --- | --- | --- |
| Valid | 10 | 1 | 1.0 |
|  | 12 | 1 | 1.0 |
|  | 16 | 1 | 1.0 |
|  | 20 | 34 | 33.7 |
|  | 23 | 5 | 5.0 |
|  | 25 | 18 | 17.8 |
|  | 28 | 1 | 1.0 |
|  | 30 | 19 | 18.8 |
|  | 35 | 5 | 5.0 |
|  | 40 | 4 | 4.0 |
|  | 44 | 1 | 1.0 |
|  | 50 | 2 | 2.0 |
|  | 60 | 1 | 1.0 |
|  | 70 | 1 | 1.0 |
|  | Total | 94 | 93.1 |
| Missing |  | 7 | 6.9 |
