# Supplementary material for: Pregnancy Outcomes in Women With Primary Adrenal Insufficiency: Data From a Multicentre Cohort Study
Source: BJOG. 2025 Mar 30;132(8):1122–9. doi: 10.1111/1471-0528.18143 (PMC12137789; doi:10.1111/1471-0528.18143)
Supplement: Supplementary file 3 — Table S2. Supporting Information. [file BJO-132-1122-s003.docx]

Table 2 Total daily dose of fludrocortisone in relation to pregnancy stage

**Total Dose (mcg/day) prepregnancy**

|  | | Frequency | Percent |
| --- | --- | --- | --- |
| Valid | 0 | 15 | 14.9 |
|  | 50 | 11 | 10.9 |
|  | 75 | 2 | 2.0 |
|  | 100 | 42 | 41.6 |
|  | 125 | 3 | 3.0 |
|  | 150 | 5 | 5.0 |
|  | 200 | 11 | 10.9 |
|  | 300 | 5 | 5.0 |
|  | 600 | 2 | 2.0 |
|  | Total | 96 | 95.0 |
| Missing |  | 5 | 5.0 |

| **Dose Fludro (mcg/day) First Trimester (0-14 weeks)** |
| --- |

|  | | Frequency | Percent |
| --- | --- | --- | --- |
| Valid | 0 | 15 | 14.9 |
|  | 50 | 9 | 8.9 |
|  | 75 | 2 | 2.0 |
|  | 100 | 37 | 36.6 |
|  | 125 | 5 | 5.0 |
|  | 150 | 7 | 6.9 |
|  | 175 | 1 | 1.0 |
|  | 200 | 14 | 13.9 |
|  | 300 | 5 | 5.0 |
|  | 600 | 2 | 2.0 |
|  | Total | 97 | 96.0 |
| Missing |  | 4 | 4.0 |

| **Dose Fludro (mcg/day) Second Trimester (15-28 weeks)** |
| --- |

|  | | Frequency | Percent |
| --- | --- | --- | --- |
| Valid | 0 | 14 | 13.9 |
|  | 50 | 9 | 8.9 |
|  | 75 | 2 | 2.0 |
|  | 100 | 32 | 31.7 |
|  | 125 | 4 | 4.0 |
|  | 150 | 12 | 11.9 |
|  | 175 | 2 | 2.0 |
|  | 200 | 14 | 13.9 |
|  | 300 | 5 | 5.0 |
|  | 350 | 1 | 1.0 |
|  | 600 | 2 | 2.0 |
|  | Total | 97 | 96.0 |
| Missing |  | 4 | 4.0 |

| **Dose Fludro (mcg/day) Third trimester (29 weeks and later)** |
| --- |

|  | | Frequency | Percent |
| --- | --- | --- | --- |
| Valid | 0 | 14 | 13.9 |
|  | 50 | 7 | 6.9 |
|  | 75 | 2 | 2.0 |
|  | 100 | 32 | 31.7 |
|  | 125 | 4 | 4.0 |
|  | 150 | 8 | 7.9 |
|  | 175 | 2 | 2.0 |
|  | 200 | 16 | 15.8 |
|  | 250 | 1 | 1.0 |
|  | 300 | 5 | 5.0 |
|  | 350 | 2 | 2.0 |
|  | 400 | 1 | 1.0 |
|  | 590 | 1 | 1.0 |
|  | 600 | 1 | 1.0 |
|  | Total | 96 | 95.0 |
| Missing |  | 5 | 5.0 |
