# Supplementary material for: Pregnancy Outcomes in Women With Primary Adrenal Insufficiency: Data From a Multicentre Cohort Study
Source: BJOG. 2025 Mar 30;132(8):1122–9. doi: 10.1111/1471-0528.18143 (PMC12137789; doi:10.1111/1471-0528.18143)
Supplement: Supplementary file 4 — Table S3. Supporting Information. [file BJO-132-1122-s001.docx]

**Table 3 Electrolyte levels through pregnancy**

| Variable | N | Mean (SD) | Median | IQR | Minimum | Maximum |
| --- | --- | --- | --- | --- | --- | --- |
| Na trimester 1 mmol/l | 66 | 135.8 (4.2) | 136 | 134-138 | 122 | 148 |
| Na trimester 2 mmol/l | 65 | 134.5 (3.8) | 135 | 133-137 | 123 | 142 |
| Na trimester 3 mmol/l | 67 | 135.2 (3.5) | 135 | 134-137 | 126 | 142 |
| K trimester 1 mmol/l | 65 | 4.33 (0.44) | 4.3 | 4.0-4.6 | 3.4 | 5.7 |
| K trimester 2 mmol/l | 65 | 4.30 (0.49) | 4.3 | 3.9-4.6 | 3.4 | 5.8 |
| K trimester 3 mmol/l | 67 | 4.31 (0.46) | 4.3 | 4.0-4.6 | 2.5 | 5.3 |
